# Case report: Primary infection of conjunctival histoplasmosis mimicking a large conjunctival neoplasm in an immunosuppressed patient

**DOI:** 10.3389/fmed.2024.1483788

**Published:** 2024-12-09

**Authors:** Jonathan Lam, Rumana Hussain, Sarah E. Coupland, Heinrich Heimann

**Affiliations:** ^1^St Pauls Eye Clinic, Liverpool University Hospitals NHS Foundation Trust, Liverpool, United Kingdom; ^2^Liverpool Ocular Oncology Research Group, Department of Eye and Vision Science, University of Liverpool, Liverpool, United Kingdom; ^3^Liverpool Clinical Laboratories, Liverpool University Hospitals NHS Foundation Trust, Liverpool, United Kingdom

**Keywords:** conjunctival tumor, histoplasmosis, immunosuppression, masquerade, amphotericin B

## Abstract

We report a rare case of a rapidly growing and large conjunctival histoplasmosis lesion in an immunosuppressed West African woman in her 80s, affecting her only eye. The patient had undergone a renal transplant and was on immunosuppressive medications. Additionally, she had previously been treated for presumed systemic histoplasmosis with itraconazole more than 5 years ago. Despite receiving previous treatment, she presented with a large conjunctival lesion, which was confirmed to be histoplasmosis after an excisional biopsy. The lesion was observed to grow over a 2-week period, causing significant visual impairment. Following a comprehensive systems review and investigation, the conjunctiva was identified as the sole site of infection. This case underscores the rarity of sight-threatening Histoplasma infections in the conjunctiva of an immunosuppressed individual. Conjunctival histoplasmosis lesions are rarely reported, with even fewer cases documented as primarily originating from this location.

## Introduction

Histoplasmosis, caused by *Histoplasma capsulatum*, is a rare ocular infection that is predominantly endemic to specific regions such as West Africa (1). While this condition typically presents as “punched-out” choroidal lesions in presumed ocular histoplasmosis syndrome (POHS) in immunocompetent individuals, its manifestation in immunosuppressed patients can lead to widespread hematogenous dissemination, including ocular involvement (2, 3). However, primary ocular histoplasmosis lesions in the conjunctiva remain rarely documented (4–6). In this study, we report a unique case of a West African woman with immunosuppression who presented with rapid growth of a large conjunctival histoplasmosis lesion, leading to blindness in her sole functioning eye over a period of 2 weeks.

## Case presentation

An elderly West African woman in her 80s was referred to our tertiary ocular oncology unit due to a suspected neoplastic lesion in her only remaining right eye. The lesion, first noticed by her daughter approximately 2 weeks earlier, had rapidly grown, causing loss of vision, pain, discomfort, and difficulty closing the eye due to its size. The patient denied any systemic symptoms, preceding illnesses, weight loss, loss of appetite, or fevers.

Her medical history included immunohistochemical staining for CD68PG highlighted cells of monocytic origin, against which the organism was distinct. In addition, she was treated for presumed systemic histoplasmosis 5 years ago, following a lip biopsy suggestive of fungal invasion and positive Histoplasma serology, with a 12-month course of itraconazole. The lip lesion resolved with this treatment, and the systemic histoplasmosis was presumed to have cleared after the therapy.

Regarding her ocular history, she was previously treated at an external facility for an undetermined bilateral cicatrizing conjunctival disease, which led to corneal decompensation and the subsequent left eye enucleation due to a non-healing ulcer infected with *Pseudomonas*. Multiple biopsies did not show any evidence of histoplasmosis or any other known cause of the cicatrizing process. As a result of the cicatrizing disease, the right eye also developed a *Pseudomonas* corneal infection, necessitating a penetrating keratoplasty. This graft failed non-infectiously, and she underwent a re-graft with the most recent penetrating keratoplasty performed 1 year before the presentation. At the time of the presentation, she received dexamethasone 0.1% drops once a day. The underlying conjunctival disease remained inactive for over a decade since its initial presentation.

**Figure 1:**
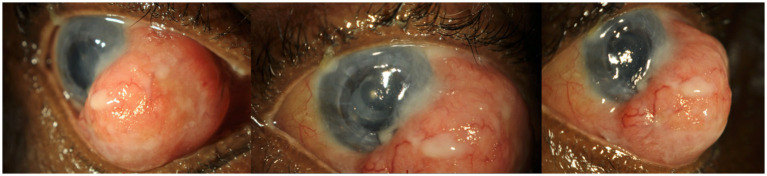
The patient’s right eye at presentation. A large, non-pigmented conjunctival mass on the bulbar conjunctiva, encompassing the 2 to 7 o’clock limbus. The surface of the lesion can be seen to be smooth, with dilated blood vessels throughout the lesion.

On examination, the patient exhibited hand movement visual acuity in her right eye. A large, solid, non-pigmented mass was observed, encompassing the 2 to 7 o’clock corneal limbus and extending nearly the full extent of the nasal bulbar conjunctiva. The surface of the mass was smooth, with dilated vessels noted on its surface, and it had a rubbery texture on palpation (Figure 1). The size of the lesion caused diplopia in the eye and impaired adduction toward the lesion.

Lagophthalmos was observed as a result of the lesion’s size. Anterior segment examination revealed a clear penetrating keratoplasty graft, quiet anterior chamber, and pseudophakic intraocular lens. Gonioscopy and posterior segment examination were challenging due to the patient’s discomfort and lack of cooperation. B-scan ultrasound did not reveal any obvious posterior pathology or deep invasion of the lesion into the eye. The contralateral left eye socket exhibited a normal-appearing conjunctiva, and the orbital implant exhibited good movements without signs of extrusion.

## Differential diagnosis

The primary working diagnosis was a rapidly progressing conjunctival neoplasm, encompassing a range of conditions categorized as ocular surface squamous neoplasias. Specifically, squamous cell carcinoma, lymphoma, and amelanotic melanoma were considered the most concerning and rapidly developing diagnoses. Other non-cancerous lesions included angiomyxoma, myxoma, and amyloidosis.

In addition, the possibility of a conjunctival granuloma of infective or inflammatory etiology was considered. These potential causes, although rare, include histoplasmosis (in light of the patient’s prior history of histoplasmosis affecting the lip), tuberculosis, and sarcoidosis. The likelihood of a foreign body granuloma was deemed less probable, given the patient’s past ocular surgical history.

## Investigation and management

The following week, the patient underwent an excisional biopsy for diagnostic and therapeutic purposes. Due to the large size of the lesion, a conjunctival autograft from the patient’s contralateral socket was performed to cover the defect left by the excision.

**Figure 2:**
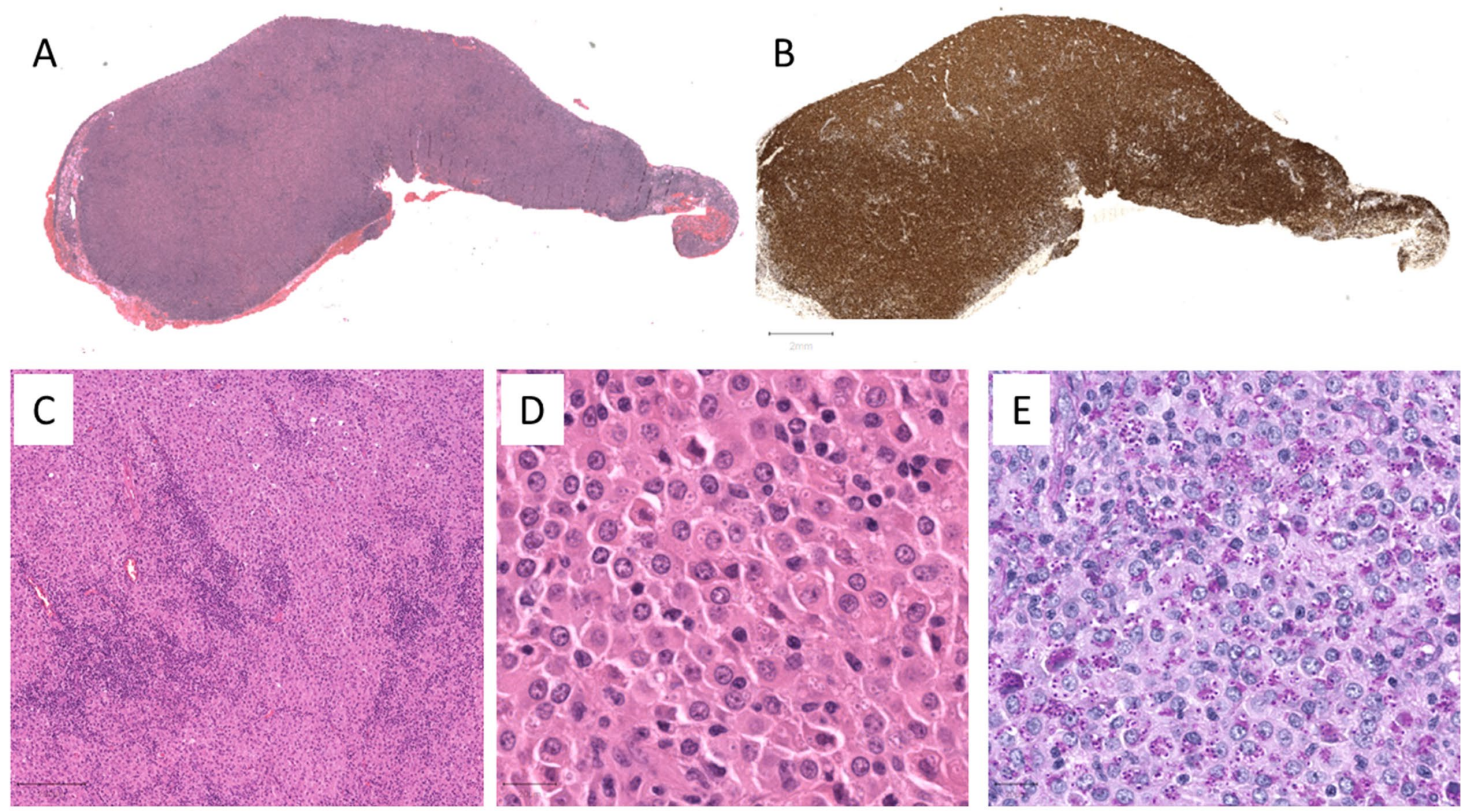
**(A)** Low power magnification of the hematoxylin and eosin (H&E) section of the conjunctival tumor mass. **(B)** Low power magnification of the immunohistochemical stain (CD68PG) highlighting the dense macrophages within the conjunctival tumor mass. **(C)** Mid-power magnification of the H&E section showing an admixture of the macrophages and lymphocytes. **(D)** High power magnification of the H&E section showing epithelioid-like macrophages with eosinophilic cytoplasm containing numerous small round-to-oval basophilic bodies surrounded by a clear halo, characteristic of *Histoplasma capsulatum*. **(E)** The D-PAS stain highlights these spore-like structures.

The histological sections demonstrated a large central tumoral mass comprising a dense infiltration of macrophages with admixed lymphocytes, along with some small foci of necrosis surrounded by palisading macrophages. Within the large pale cytoplasm of the macrophages were numerous small round-to-oval basophilic bodies surrounded by a clear halo, which is a characteristic feature of *Histoplasma capsulatum*. These were highlighted in the periodic acid-Schiff (PAS) with diastase (PAS-D) stain, while immunohistochemical staining for CD68PG highlighted cells of monocytic origin, against which the organism was distinct. The foci of necrosis were identified (Figure 2), but the Ziehl–Neelsen staining did not reveal additional mycobacteria.

The patient was referred to the infectious disease unit for care. Systemic treatment of itraconazole was prescribed for inpatient use, based on a presumed systemic hematogenous spread, with careful monitoring of renal function. Amphotericin B eye drops (0.15%) were administered to the treated eye four times a day for 4 weeks. Dexamethasone 0.1% eye drops, initially prescribed for maintaining the health of her corneal graft, were discontinued for the first 2 weeks following the excision to mitigate the risk of exacerbating any residual fungal infection. Subsequently, they were reintroduced at a once-daily dosage, with close monitoring to detect any signs of recurrence (Table 1).

**Table 1:** Summary of previously reported cases of conjunctival histoplasmosis.

Case report	Location of lesion	Size	Appearance	Speed of onset	Treatment	Recurrence?
Behera et al. (4)	Nasal bulbar conjunctiva involving corneal limbus	18x12mm	Non-pigmented, discreet mass with dilated vessels within it. ‘Freely mobile’ with no scleral attachment	2 months	Excisional biopsy alone	Nil after 9 months
Knox et al. (5)	Nasal bulbar conjunctiva involving medial rectus	8x8mm	Small, solitary, inflamed with surrounding reactive episcleral vessels, non-pigmented. Adherent to the underlying tissues, including the medial rectus.	Unknown	Excisional biopsy alone	Nil after 12 years
Pujari et al. (6)	Temporal bulbar conjunctiva	Unspecified, but involving the extent of the visible temporal intra-palpebral bulbar conjunctiva	Diffuse, non-pigmented conjunctival thickening replacing healthy tissue	4 months	Intravenous Amphotericin B	Patient died from comorbid indirect causes several weeks after the initiation of treatment

**Figure 3:**
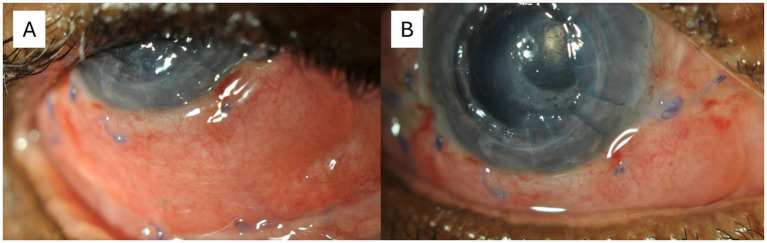
The eye 6 weeks post-excisional biopsy. **(A)** The eye in upgaze. **(B)** The eye in straight gaze. The conjunctival autograft from the contralateral socket can be seen to cover the excised area. As can be seen, the graft was taken and the Vicryl sutures remained *in situ*. There was no obvious recurrence in the excised area or surrounding tissue. There were two pre-existing corneal nylon sutures in the patient’s penetrating keratoplasty.

During the 6-week inpatient stay at our institution, there was no recurrence of the lesion. The grafted conjunctival area healed well (Figure 3). Notably, her visual acuity improved to 20/200, and a fundus examination was sufficiently clear, revealing no abnormalities. Subsequently, she was transferred to a district hospital closer to her residence for ongoing systemic itraconazole therapy and renal monitoring.

Unfortunately, despite receiving treatment, the patient’s overall health deteriorated, without any discernible direct cause, such as renal failure related to the treatment. Consequently, she transitioned to palliative care. Due to her declining health status, further ophthalmic follow-up was not possible.

## Discussion

Histoplasmosis affecting the eye, particularly outside the retina/choroid, has been rarely documented. Among the three other reported cases of conjunctival Histoplasma lesions, all occurred in individuals who were either immunosuppressed or immunocompromised (4–6). Notably, only one case report documented a primary lesion originating from the conjunctiva (6). However, none of these previously reported cases exhibited a lesion as extensive, aggressive, or rapidly growing as observed in our patient’s case. The size and aggressiveness of the lesion posed a significant threat to the patient’s vision, necessitating a prompt therapeutic and diagnostic excisional biopsy. In addition, none of the previous reports documented the disease in a patient who had also been previously treated for presumed systemic histoplasmosis dissemination. Given this medical history, the possibilities include that the current conjunctival infection represents a new Histoplasma infection or a reactivation of latent systemic disease.

An excisional biopsy was proven effective as a primary treatment modality in two reported cases, resulting in complete resolution of the Histoplasma lesion without recurrence (4, 5). However, in the context of ocular involvement, it is assumed that Histoplasma has disseminated hematogeneously after initial pulmonary inhalation. In such cases, systemic antifungal therapy, such as itraconazole or amphotericin B, may be warranted (6). This approach was particularly relevant in our case, given the patient’s prior history of presumed systemic histoplasmosis, confirmed by a lip biopsy and Histoplasma serology, for which she underwent a full course of itraconazole treatment. Close monitoring of renal function was essential during the inpatient care due to the nephrotoxicity associated with itraconazole, compounded by the patient’s history of renal transplant.

In addition, local topical therapy was performed using amphotericin B 0.15% eye drops to either treat or prevent further Histoplasma conjunctival infection following an excisional biopsy (6). We incorporated this therapy into our patient’s treatment regimen and opted to temporarily discontinue her use of topical dexamethasone 0.1% eye drops to minimize the risk of recurrence. Once we were satisfied with the absence of early recurrence, we resumed the administration of dexamethasone drops to prevent the rejection of the patient’s existing corneal graft.

Our case highlights the challenge posed by an aggressive conjunctival histoplasmosis lesion, which may be a recurrence or an inadequately cleared systemic histoplasmosis infection, even after prior treatment with itraconazole.

## Data Availability

The original contributions presented in the study are included in the article/supplementary material, further inquiries can be directed to the corresponding author/s.
